# Nurses who work in rural and remote communities in Canada: a national survey

**DOI:** 10.1186/s12960-017-0209-0

**Published:** 2017-05-23

**Authors:** Martha L. P. MacLeod, Norma J. Stewart, Judith C. Kulig, Penny Anguish, Mary Ellen Andrews, Davina Banner, Leana Garraway, Neil Hanlon, Chandima Karunanayake, Kelley Kilpatrick, Irene Koren, Julie Kosteniuk, Ruth Martin-Misener, Nadine Mix, Pertice Moffitt, Janna Olynick, Kelly Penz, Larine Sluggett, Linda Van Pelt, Erin Wilson, Lela Zimmer

**Affiliations:** 1 School of Nursing, University of Northern British Columbia, 3333 University Way, Prince George, BC V2N 4Z9 Canada; 2 College of Nursing, University of Saskatchewan, Health Sciences Building, E Wing, 104 Clinic Place, Saskatoon, SK S7N 2Z4 Canada; 3 Faculty of Health Sciences, University of Lethbridge, 4401 University Dr W, Lethbridge, AB T1K 6T5 Canada; 4 Northern Health Authority, 510-1488 4th Avenue, Prince George, BC V2L 4Y2 Canada; 5 Department of Geography, School of Health Sciences and Northern Medical Program, University of Northern British Columbia, 3333 University Way, Prince George, BC V2N 4Z9 Canada; 6 University of Saskatchewan, 104 Clinic Place, P.O. Box 23, Saskatoon, SK S7N 2Z4 Canada; 7 Centre de Reserche, Hôpital Maisonneuve-Rosemont, Université de Montréal, 5415 Assomption Boulevard, Montreal, QC H1T 2M4 Canada; 8 School of Nursing, Laurentian University, 935 Ramsey Lake Rd, Sudbury, ON P3E 2C6 Canada; 9 School of Nursing, Dalhousie University, 5869 University Ave, Halifax, NS B3H 4R2 Canada; 10 North Slave Research Centre, Aurora College, Bag 9700, 5004-54th St, Yellowknife, NT X1A 2R3 Canada; 11 College of Nursing, University of Saskatchewan, 4400 4th Avenue, Regina, SK S4T 0H8 Canada

**Keywords:** Rural, Remote, Northern, Workforce, Nurses, Recruitment, Retention, Methodological challenges, Survey, Canada

## Abstract

**Background:**

In Canada, as in other parts of the world, there is geographic maldistribution of the nursing workforce, and insufficient attention is paid to the strengths and needs of those providing care in rural and remote settings. In order to inform workforce planning, a national study, *Nursing Practice in Rural and Remote Canada II*, was conducted with the rural and remote regulated nursing workforce (registered nurses, nurse practitioners, licensed or registered practical nurses, and registered psychiatric nurses) with the intent of informing policy and planning about improving nursing services and access to care. In this article, the study methods are described along with an examination of the characteristics of the rural and remote nursing workforce with a focus on important variations among nurse types and regions.

**Methods:**

A cross-sectional survey used a mailed questionnaire with persistent follow-up to achieve a stratified systematic sample of 3822 regulated nurses from all provinces and territories, living outside of the commuting zones of large urban centers and in the north of Canada.

**Results:**

Rural workforce characteristics reported here suggest the persistence of key characteristics noted in a previous Canada-wide survey of rural registered nurses (2001-2002), namely the aging of the rural nursing workforce, the growth in baccalaureate education for registered nurses, and increasing casualization. Two thirds of the nurses grew up in a community of under 10 000 people. While nurses’ levels of satisfaction with their nursing practice and community are generally high, significant variations were noted by nurse type. Nurses reported coming to rural communities to work for reasons of location, interest in the practice setting, and income, and staying for similar reasons. Important variations were noted by nurse type and region.

**Conclusions:**

The proportion of the rural nursing workforce in Canada is continuing to decline in relation to the proportion of the Canadian population in rural and remote settings. Survey results about the characteristics and practice of the various types of nurses can support workforce planning to improve nursing services and access to care.

**Electronic supplementary material:**

The online version of this article (doi:10.1186/s12960-017-0209-0) contains supplementary material, which is available to authorized users.

## Background

In 2015, 11.8% (45,926) of regulated nurses in Canada’s 10 provinces provided care for the 17.4% of the population living in rural or remote areas of the provinces [1]. The geographic imbalance of nurses in Canada between rural and urban communities mirrors that of many other countries worldwide [2–5]. In Canada, as elsewhere, persistent challenges in ensuring a well-qualified nursing workforce in rural and remote communities have impacted health services and patient outcomes [2, 6–10].

Despite the increased attention globally to recruitment and retention of health professionals, including nurses (e.g., [3, 11]), there has been less emphasis on recruitment and retention policies and practices that can be effective in rural areas [7, 12]. Greater organizational, regional, and country-related context-sensitivity is needed to determine effective recruitment and retention approaches [11, 13, 14]. In order to create such sensitivity to the needs of the nursing workforce in rural and remote contexts, where nurses are sometimes the only health care providers, more knowledge about that workforce, its diversity, and needs is urgently required.

Four types of nurses comprise the Canadian regulated nursing workforce. Registered nurses (RNs) and nurse practitioners (NPs) practice in all ten provinces and three territories. Practical nurses are referred to as licensed practical nurses (LPNs) across Canada, except in the province of Ontario (ON), where they are referred to as registered practical nurses. There are registered psychiatric nurses (RPNs) only in the four western provinces: British Columbia (BC), Alberta (AB), Saskatchewan (SK), and Manitoba (MB), and the three northern territories: Nunavut (NU), Northwest Territories (NT), and the Yukon (YT).

In the early 2000s, a national study, *The Nature of Nursing Practice in Rural and Remote Canada* (*RRNI*), described the rural and remote registered nursing workforce for the first time [15–18]. The study’s multifaceted analysis included depictions of predictors of intent to leave [19], practicing alone [20], gender issues [21], rural Aboriginal nurses [22], barriers to information use [23], barriers to continuing education [24], predictors of job satisfaction [25], working in remote primary care [26], being a rural nurse [27], professionalism in rural nursing [28], rural community RNs’ community attachment and satisfaction [29], and how rural RNs define rurality [30]. Other Canadian studies have identified factors to support registered nurses and practical nurses in rural acute care hospitals [31], the fit of rural policy to the rural nursing workforce [32], practice issues for rural obstetrical nurses [33], and retention issues for rural practical nurses [34]. Although there are calls for increased attention to skill mix within the rural health workforce [35], few studies in Canada or elsewhere have delineated and compared the characteristics and practice of various types of rural nurses. Such delineation is necessary in order that workforce planning can be suitably tailored and all types of nurses can be appropriately supported. The overall questions guiding the *Nursing Practice in Rural and Remote Canada II* (*RRNII*) study were: What is the nature of nursing practice in rural and remote Canada? How can the capacity of nursing services and access to nursing care in rural and remote Canada be enhanced? The purpose of this article is to describe the study methods and examine the characteristics of the rural and remote nursing workforce with a focus on important variations among nurse types and regions.

## Methods

### Questionnaire development

The questionnaire used in the RRNI survey (2001–2004) was used as a base, with a conceptual framework and research objectives (Additional file 1: Figure S1 and Additional file 2: Table S1) guiding questionnaire development. The conceptual framework was created by the Research Team based on an integrated view of workforce planning [36] and reflected the interconnectedness of individual, workplace, community characteristics, and nursing practice in rural and remote settings. The research objectives concerned five priority areas: (1) nursing roles and functions, including engagement of nurses with primary health care (PHC); (2) recruitment; (3) retention; (4) preparedness for practice; and (5) implications for knowledge translation and policy. The content domains were individual (demographics and employment), work community (population, travel, resources, engagement, sense of community), workplace (engagement in primary health care), nursing practice (scope, employment patterns, organizational commitment, information/education sources, interprofessional practice, job resources and demands, violence), and personal (health, burnout, stress).^1^

The questionnaire was translated from English to French, adapted, and back translated by qualified translators. Bilingual researchers and advisors reviewed the translation to ensure conceptual, item, and semantic equivalence [37].

### Pilot test of questionnaire

In the first of two pilot tests, the 42-page (46 pages in French) questionnaire was sent in paper and online to Research Team members, to forward to nurses with recent experience working in rural or remote areas. As few responses were received, a second 15-page pilot survey, distributed through snowball sampling, was implemented in paper and online with 89 nurses in order to psychometrically test the three newly developed scales [38]. The questionnaire was refined to 27 pages (31 pages in French) by the 16-member Research Team, with feedback from the 19-member Advisory Team (nursing leaders and policy-makers from all provinces and territories).

### Sampling frame

Eligible participants were regulated nurses currently employed in nursing in a rural or remote area or on leave for 6 months or less. The number of nurses sampled was derived from an analysis of the 2010 Canadian Institute for Health Information Nurses Database [39]. A multi-level systematic sample was used, and the sampling frame included (1) RNs, NPs, LPNs, and RPNs with rural postal code sets within each of the 10 Canadian provinces; (2) all rural and remote NPs; and (3) all nurses (RNs, NPs, LPNs, and RPNs) working in the territories (Yukon Territory, Northwest Territory, and Nunavut). The sampling frame was designed with the goal of achieving statistically significant (confidence level of 95% and a margin of error of 0.05) results provincially, territorially, and nationally.

We used Statistics Canada’s definition of Rural and Small Town Canada [40]. “Rural” refers to communities with a core population of less than 10 000 people, where less than 50% of the employed population commutes to larger urban centers for work. Remote and northern places are included in the Rural and Small Town definition [40]. Due to the small numbers of nurses registered in the three northern territories, we included all nurses in the territories, even those in Yellowknife, NT, and Whitehorse, YT, with populations of approximately 20 000 and 23 000, respectively. Our use of the terms, rural and remote, reflect Canadian nurses’ use in everyday language and perceptions [30, 41].

### Participant sampling

The multi-level stratified systematic sample was obtained using three levels of stratification. The first level was by province/territory, although the territories were a special case (sampled 100%). The second level of stratification was by type of nurse. We achieved the first two levels by selecting participants through the provincial/territorial nursing associations for each regulated nurse type. Further stratification by geographic area within provinces was done to allow for a reasonably representative geographic distribution of rural nurses, using rural postal codes (Statistics Canada’s 2009 Postal Code Conversion File) in which the first three characters represent the forward sortation areas (FSA) across regions of the province.

Each nursing association was given an excel file with all rural postal codes in their province. The associations matched the rural postal codes to their nurses’ work postal code (or home postal code, if work postal code is not available), sorted the list alphanumerically by postal code, and then conducted a systematic sample by dividing the total number of rural nurses in the association database by the desired sample size. Using the resultant number “*k*,” every *k*th rural nurse on the list was selected until the sample size was reached. This method of sampling preserved randomness but provided a sample with greater distribution across the geographical region than simple random sampling would have done.

The stratified systematic samples were augmented by 30%, which was the value deemed necessary from the *RRNI* survey to maintain the minimum sample size accounting for non-return, ineligible responses, and duplicate mailings.

All nursing associations participated; however, four of the 29 nursing associations were unable to attain the requested sample size. Among the reasons were the following: nursing association members whose workplace or home postal codes matched those on the list had retired and were not working (and thus were not eligible to participate); some nurses did not indicate consent to be included in research; consolidation/closing of rural health care workplaces had occurred; and postal code files may have been incomplete.

### Survey implementation

The survey was implemented between April 2014 and August 2015 using the Dillman et al. method for maximizing survey returns, including recommended formatting of the questionnaire, offering participants an incentive (draw for an iPad), and sending persistent follow-ups [42]. The survey packages included a survey booklet, cover letter, information sheet, username for online response, and self-addressed, postage paid return envelope.

The survey and reminders were mailed to the participants by either their nursing association or the Research Hub at the University of Northern British Columbia. There were three methods of survey distribution: mail-out by 21 nursing association direct to nurses’ home addresses, mail-out by the Research Hub direct to nurses’ home addresses confidentially provided by six nursing associations, and mail-out by the Research Hub to nurses’ workplaces in one province and one territory. In these two mail-outs, the associations facilitated connections with the workplaces but were not able to undertake the mail-out or to provide participants’ names and addresses. As a result, in these two mail-outs, it was not possible to track participants or to identify non-respondents.

Persistent follow-up was achieved by sending up to four mail-outs over a 5-week period: an initial survey package, first reminder postcard, second reminder postcard, and a replacement survey package. The participants were invited to return the paper survey or to respond online.

### Data management

Data were entered using FileMaker Pro Advanced 13. All participant comments were entered verbatim. French responses to “other” questions were translated into English prior to data entry and qualitative data from two open-ended questions were entered in the language in which they were submitted (English or French). Double entry was performed for 13% of the surveys; inconsistencies between entries for the same case and errant values were identified and resolved by assessing frequencies and checking the original questionnaire.

### Data analysis

Data were analyzed in IBM SPSS Statistics 23 with frequencies and one-way analyses of variance (ANOVA) presented in this analysis. Significance level was set at 0.05 for analyses of association between satisfaction (primary work community and nursing practice) and region and nurse type. A Bonferroni-adjusted alpha level was used to reduce the probability of type 1 errors when conducting multiple tests. When Levene’s test of homogeneity of variances was satisfied, Tukey’s honestly significant difference (HSD) post hoc comparisons were used to follow up significant effects and confirm where differences existed between groups. When Levene’s test was not satisfied, the Welch ANOVA statistic was reported and Games-Howell post hoc tests were conducted to follow up significant ANOVA results.

### Ethics

Approval was received from the research ethics boards of the researchers’ six universities and the three territories’ research access organizations. Approval processes for survey implementation were negotiated in detail with each of the 29 nursing associations, and the two workplaces to conform to each organization’s protocols and processes.

## Results

### Response rates

The initial target sample included 10 072 RNs, NPs, LPNs, and RPNs across Canada. Of 9622 eligible participants, 3822 returned completed surveys, for a response rate of 40% (3822/9622). Of these, 728 (19%) were completed online and 3094 (81%) on paper.

The response rate by nurse type for all of Canada was RNs 40%, NPs 58%, LPNs 38%, and RPNs 38%. The response rate by province/territory was Nova Scotia (NS) 47%; Manitoba (MB) 47%; Yukon (YT) 46%; Ontario (ON) 44%; Saskatchewan (SK) 43%; New Brunswick (NB) 39%; Newfoundland and Labrador (NL) 38%; British Columbia (BC) 38%; Northwest Territories and Nunavut combined (NT/NU) (RNs and NPs only) 37%; Quebec (QC) 35%; Alberta (AB) 34%; Northwest Territories (NT) (LPNs only) 30%; Prince Edward Island (PE) 26%; and Nunavut (NU) (LPNs only) 21%.

### Characteristics of nurses working in rural and remote settings

Of the 3822 respondents, 163 were NPs, 2082 were RNs, 1370 were LPNs, and 207 were RPNs. Nearly 80% of the survey respondents were married or living with a partner. Fewer than 7% of the respondents were of First Nations, Inuit, or Métis ancestry, and 43.7% of the respondents had one or more dependent children living with them.

Table 1 2 indicates the age and gender distribution of nurses by province and territories. Nationally, the majority of respondents were female (93.6%). The gender breakdown across nurse type showed some variance, although females accounted for the majority: 96.2% of NPs, 93.8% of RNs, 94.4% of LPNs, and 85% of RPNs were female. More than 60% of the respondents were 45 years of age or older and less than 20% were age 34 years or younger. The largest percentage of NPs (36.5%), LPNs (30.3%), and RPNs (34.0%) were between 45 and 54 years old, and most commonly, RNs were between 55 and 64 years old (29.6%).

**Table 1:** Age group and gender by region of primary employment

Age group	Atlantic (NL, NS, NB, PE) *n* (%)	QC *n* (%)	ON *n* (%)	MB/SK *n* (%)	AB/BC *n* (%)	Territories (YT, NT, NU) *n* (%)	Total *N* (%)
<25	18 (2.1)	20 (7.0)	–	16 (2.1)	12 (2.1)	–	73 (2.2)
25–34	122 (14.1)	77 (27.0)	44 (11.5)	113 (15.2)	112 (19.4)	114 (21.8)	582 (17.2)
35–44	176 (20.3)	67 (23.5)	71 (18.5)	133 (17.9)	99 (17.2)	111 (21.2)	657 (19.4)
45–54	283 (32.7)	76 (26.7)	124 (32.4)	219 (29.4)	158 (27.4)	124 (23.7)	984 (29.1)
55–64	231 (26.7)	40 (14.0)	127 (33.2)	229 (30.7)	175 (30.3)	136 (26.0)	938 (27.8)
>64	36 (4.2)	5 (1.8)	14 (3.7)	35 (4.7)	21 (3.6)	35 (6.7)	146 (4.3)
Female subtotal	882 (94.4)	288 (93.8)	397 (97.5)	760 (93.5)	589 (93.5)	539 (90.0)	3 455 (93.6)
<25	–	–	–	–	–	–	–
25–34	10 (19.2)	–	–	8 (16.0)	5 (12.5)	10 (16.9)	40 (17.4)
35–44	13 (25.0)	–	–	11 (22.0)	5 (12.5)	23 (39.0)	59 (25.7)
45–54	18 (34.6)	–	–	16 (32.0)	17 (42.5)	9 (15.3)	65 (28.3)
55–64	10 (19.2)	7 (36.8)	–	13 (26.0)	10 (25.0)	16 (27.1)	59 (25.7)
>64	–	–	–	–	–	–	5 (2.2)
Male subtotal	52 (5.6)	19 (6.2)	10 (2.5)	53 (6.5)	41 (6.5)	60 (10.0)	235 (6.4)
<25	19 (2.1)	20 (6.6)	–	16 (2.0)	13 (2.1)	–	75 (2.1)
25–34	132 (14.3)	81 (26.6)	47 (11.9)	121 (15.2)	118 (19.0)	124 (21.2)	623 (17.2)
35–44	190 (20.6)	71 (23.3)	74 (18.7)	145 (18.2)	104 (16.8)	135 (23.0)	719 (19.8)
45–54	304 (32.9)	81 (26.6)	125 (31.6)	235 (29.5)	176 (28.4)	133 (22.7)	1 054 (29.1)
55–64	243 (26.3)	47 (15.4)	132 (33.4)	243 (30.5)	186 (30.0)	153 (26.1)	1004 (27.7)
>64	36 (3.9)	5 (1.6)	14 (3.5)	37 (4.6)	23 (3.7)	37 (6.3)	152 (4.2)
Total sample	969 (25.4)	314 (8.2)	422 (11.0)	841 (22.0)	655 (17.1)	621 (16.2)	3 822 (100.0)

As shown in Table 2, many respondents held more than one education credential. Although most respondents held education credentials in nursing (*n* = 3725; 97.5%), a subset (*n* = 278; 7.3%) held non-nursing credentials either in addition to, or instead of, nursing credentials. The most commonly attained non-nursing credential was a Bachelor’s degree, held by 5.8% of respondents. Across Canada, 50.2% of RNs held a Bachelor’s degree in Nursing and 15.3% of NPs held Master’s degrees in Nursing. Diplomas were the most common nursing education credential among RNs, LPNs, and RPNs. NPs were most likely (68.1%) to hold a Bachelor’s degree in Nursing, while Diplomas in Nursing were held most frequently by RNs (60%), LPN/RPN Diplomas by LPNs (97.6%), and Diplomas in Psychiatric Nursing by RPNs (79.5%).

**Table 2:** Education credentials by region of primary employment

Education credentials	Atlantic *n* (%)	QC *n* (%)	ON *n* (%)	MB/SK *n* (%)	AB/BC *n* (%)	Territories *n* (%)	Total *N* (%)
LPN Diploma	451 (46.9)	127 (40.4)	193 (46.2)	326 (39.1)	261 (40.1)	102 (16.6)	1 460 (38.5)
LPN Equivalency	13 (1.4)	6 (1.9)	11 (2.6)	19 (2.3)	13 (2.0)	–	66 (1.7)
Diploma in Nursing	303 (31.5)	144 (45.9)	175 (41.9)	258 (30.9)	204 (31.3)	274 (44.7)	1 358 (35.8)
Diploma in Psych Nursing	5 (0.5)	–	–	119 (14.3)	68 (10.4)	10 (1.6)	206 (5.4)
Advanced Diploma in Psych Nursing	–	–	–	5 (0.6)	–	–	15 (0.4)
Bachelor’s in Nursing	284 (29.6)	98 (31.2)	83 (19.9)	202 (24.2)	191 (29.3)	318 (51.9)	1 176 (31.0)
Post Basic Certificate	96 (10.0)	20 (6.4)	40 (9.6)	32 (3.8)	69 (10.6)	140 (22.8)	397 (10.5)
Rural and Remote Certificate	–	–	–	–	18 (2.8)	39 (6.4%)	69 (1.8)
NP Diploma/Certificate	15 (1.6)	6 (1.9)	26 (6.2)	18 (2.2)	5 (0.8)	19 (3.1%)	89 (2.3)
Master’s in Nursing	24 (2.5)	–	13 (3.1)	10 (1.2)	25 (3.8)	29 (4.7)	103 (2.7)
Doctorate in Nursing	–	–	–	–	–	–	6 (0.02)
Nursing—total sample	952	307	415	810	638	603	3 725
Bachelor’s (non-nursing)	41 (4.3)	8 (2.5)	19 (4.5)	47 (5.6)	35 (5.4)	70 (11.4)	220 (5.8)
Master’s (non-nursing)	12 (1.2)	6 (1.9)	8 (1.9)	7 (0.8)	15 (2.3)	24 (3.9)	72 (1.9)
Doctorate (non-nursing)	–	–	–	–	–	–	–
Non-nursing—total sample	50	13	25	54	48	88	278

Data reflects all credentials achieved, not the highest credential or credential respondent is currently practicing under. Percentages will not add to 100

### Employment status and work setting of rural and remote nurses

Nurses were likely to be working in either a full-time (FT) (53.6%) or part-time (PT) (30.6%) permanent position. Variations in nursing employment status were evident across regions, as shown in Table 3.

**Table 3:** Employment status by region of primary employment

Nursing employment status	Atlantic *n* (%)	QC *n* (%)	ON *n* (%)	MB/SK *n* (%)	AB/BC *n* (%)	Territories *n* (%)	Total *N* (%)
FT permanent	625 (65.6)	162 (52.1)	257 (61.5)	391 (46.8)	255 (39.2)	337 (54.9)	2 027 (53.6)
PT permanent	203 (21.3)	135 (43.4)	130 (31.1)	317 (38.0)	282 (43.3)	92 (15.0)	1 159 (30.6)
Job share	5 (0.5)	–	–	14 (1.7)	–	17 (2.8)	43 (1.1)
Casual	129 (13.5)	10 (3.2)	43 (10.3)	154 (18.4)	135 (20.7)	127 (20.7)	598 (15.8)
Contract/term	23 (2.4)	5 (1.6)	8 (1.9)	25 (3.0)	22 (3.4)	89 (14.5)	172 (4.5)
Total sample	953	311	418	835	651	614	3 782

Nursing employment status varied considerably across nurse type, such that NPs were most frequently (75.8%) employed in a FT permanent position, followed by RPNs (62.7%). This percentage was substantially lower (around 52%) for RNs and LPNs. Of all nurse types, LPNs were most likely to be employed in PT permanent positions (34.7%), and RNs and LPNs in casual nursing positions (16.5%). See Additional file 3: Table S2.

The respondents noted over 16 primary places of employment (Table 4). Nurses most commonly worked in a hospital setting (42.0%), followed by a nursing home/long-term care facility (20.6%). Considerable regional variation in primary place of employment was apparent, as indicated in Table 4.

**Table 4:** Primary place of employment by region of primary employment

Primary place of employment	Atlantic *n* (%)	QC *n* (%)	ON *n* (%)	MB/SK *n* (%)	AB/BC *n* (%)	Territories *n* (%)	Total *N* (%)
Hospital	465 (48.7)	133 (42.9)	223 (53.2)	282 (33.9)	291 (45.0)	192 (31.3)	1 586 (42.0)
Nursing home/long-term care facility	232 (24.3)	37 (11.9)	79 (18.9)	231 (27.8)	139 (21.5)	61 (10.0)	779 (20.6)
Community health center	60 (6.3)	57 (18.4)	24 (5.7)	64 (7.7)	46 (7.1)	200 (32.6)	451 (11.9)
Home care agency	42 (4.4)	6 (1.9)	21 (5.0)	50 (6.0)	30 (4.6)	17 (2.8)	166 (4.4)
Public health unit	35 (3.7)	–	9 (2.1)	22 (2.6)	33 (5.1)	27 (4.4)	127 (3.4)
Physician’s office	30 (3.1)	18 (5.8)	34 (8.1)	12 (1.4)	20 (3.1)	9 (1.5)	123 (3.3)
Mental health/crisis center	14 (1.5)	–	10 (2.4)	52 (6.3)	37 (5.7)	9 (1.5)	122 (3.2)
Integrated facility	15 (1.6)	31 (10.0)	5 (1.2)	54 (6.5)	8 (1.2)	6 (1.0)	119 (3.2)
Educational institution	23 (2.4)	6 (1.9)	–	6 (0.7)	10 (1.5)	16 (2.6)	61 (1.6)
Multidisciplinary primary health care clinic	15 (1.6)	–	–	20 (2.4)	6 (0.9)	8 (1.3)	56 (1.5)
Professional association/government	7 (0.7)	–	–	11 (1.3)	–	31 (5.1)	55 (1.5)
Other	6 (0.6)	–	–	10 (1.2)	7 (1.1)	27 (4.4)	54 (1.4)
Private nursing/self-employed	6 (0.6)	–	–	6 (0.7)	7 (1.1)	5 (0.8)	31 (0.8)
Rehab/convalescent centre	–	5 (1.6)	–	7 (0.8)	–	–	19 (0.5)
Occupational health	–	–	–	–	–	–	13 (0.3)
Nurse practitioner-led clinic	–	–	–	–	–	–	13 (0.3)
Total sample	955	310	419	832	646	613	3 775

There was also variation in primary place of employment across nurse type, in that NPs most frequently worked in community health centers (29.4%) and physician’s offices (28.8%), RNs in hospitals (45.6%) and community health centers (17.1%), LPNs in hospitals (43.8%) and nursing homes/long-term care facilities (37.1%), and RPNs in mental health/crisis centers (35.0%) and nursing homes/long-term care facilities (22.2%).

### Living and working in rural communities

A majority of the respondents (86.2%) reported their primary work community was medium to large sized, with only 13.7% of the respondents working in a small rural/remote community (Table 5). NPs, RNs, and LPNs were most likely to work in medium-sized communities (ranging from 53.2% to 58.8%), while RPNs most commonly (45.5%) worked in large communities. The primary work community was identified as being only accessible by plane by 8.4% of the respondents, with the majority of those being nurses employed in the territories (35.7%). A small segment of NPs (10.0%) and RNs (12.4%) worked in fly-in communities.

**Table 5:** Community-related variables by region of primary employment

	Atlantic *n* (%)	QC *n* (%)	ON *n* (%)	MB/SK *n* (%)	AB/BC *n* (%)	Territories *n* (%)	Total *N* (%)
Population of childhood community							
Small (999 or less)	391 (41.6)	75 (28.4)	92 (22.7)	448 (54.4)	167 (26.1)	122 (20.4)	1 295 (34.9)
Medium (1 000–9 999)	385 (41.0)	118 (39.1)	178 (43.8)	196 (23.8)	231 (36.0)	129 (21.5)	1 237 (33.3)
Large (>10 000)	163 (17.4)	109 (36.1)	136 (33.5)	180 (21.8)	243 (37.9)	348 (58.1)	1 179 (31.8)
Total sample	939	302	406	824	641	599	3 711
Population of primary work community							
Small (999 or less)	95 (10.2)	19 (6.3)	18 (4.5)	213 (26.0)	49 (7.7)	113 (18.9)	507 (13.7)
Medium (1 000-9 999)	542 (58.5)	173 (57.3)	268 (66.3)	408 (49.8)	421 (65.9)	215 (35.9)	2 027 (54.9)
Large (>10 000)	290 (31.3)	110 (36.4)	118 (29.2)	198 (24.2)	169 (26.4)	271 (45.1)	1156 (31.3)
Total sample	927	302	404	819	639	599	3 690
Live in primary work community							
Yes	495 (52.2)	183 (59.4)	239 (58.2)	413 (49.8)	397 (61.7)	429 (71.1)	2 156 (57.6)
Total sample	948	308	411	830	643	603	3 743

Nearly 58% of the respondents indicated that they live in their primary work community. RNs (60.1%) and RPNs (61.1%) were most likely to live in their primary work community, while LPNs were least likely (53.3%).

There was a relatively even split between respondents who grew up in small-, medium-, and large-sized rural and remote communities. Regional variation in size of childhood community was evident, as shown in Table 5.

As shown in Table 6, satisfaction with primary work community was significantly related to nurse type,^3^ *F*(1, 3765) = 20.52, *p* < .001, *η* ^2^ = .01, such that NPs and RNs had significantly higher mean satisfaction scores (*M* = 4.08, SD = 0.75) than LPNs and RPNs (*M* = 3.97, SD = 0.78). However, work community satisfaction did not significantly vary by region of employment, *F*_Welch_(5, 1431.70) = 0.58, *p* = .714, *η* ^2^ = .00.

**Table 6:** Satisfaction with primary work community by region of primary employment and nurse type

Overall, I am satisfied with my primary work community	Atlantic *M* (SD) *n* = 958	QC *M* (SD) *n* = 307	ON *M* (SD) *n* = 417	MB/SK *M* (SD) *n* = 833	AB/BC *M* (SD) *n* = 645	Territories *M* (SD) *n* = 607	Total *M* (SD) *n*=
NP *n* = 162	4.10 (0.70)	4.25 (0.79)	4.06 (0.81)	4.32 (0.48)	4.00 (0.63)	4.23 (0.63)	4.16 (0.69)
RN *n* = 2 045	4.10 (0.78)	4.08 (0.63)	4.08 (0.72)	4.09 (0.70)	4.07 (0.83)	4.05 (0.77)	4.08 (0.75)
LPN *n* = 1 356	3.98 (0.78)	3.88 (0.72)	4.05 (0.79)	3.98 (0.70)	3.92 (0.85)	3.97 (0.85)	3.97 (0.78)
RPN *n* = 204	–	–	–	3.97 (0.78)	3.94 (0.82)	4.33 (0.82)	3.97 (0.79)
Total sample *N* = 3 767	4.04 (0.78)	4.02 (0.69)	4.07 (0.76)	4.03 (0.71)	4.00 (0.83)	4.06 (0.78)	4.04 (0.76)

Likert scale had a range of 1–5, with a higher number representing greater satisfaction

Satisfaction with current nursing practice varied significantly across nurse type, *F*_Welch_(1, 2994.19) = 50.48, *p* < .001, *η* ^2^ = .01 (Table 7). NPs and RNs had significantly higher mean satisfaction scores (*M* = 4.01, SD = 0.79) than LPNs and RPNs (*M* = 3.80, SD = 0.87). However, satisfaction did not significantly vary by region of primary employment, *F*_Welch_(5, 1394.22) = 3.13, *p* = .008, *η* ^2^ = .00.

**Table 7:** Satisfaction with Current Nursing Practice by Region of Primary Employment and Nurse Type

Overall, I am satisfied with my current nursing practice	Atlantic *M* (SD) *n* = 909	QC *M* (SD) *n* = 306	ON *M* (SD) *n* = 400	MB/SK *M* (SD) *n* = 796	AB/BC *M* (SD) *n* = 618	Territories *M* (SD) *n* = 581	Total *M* (SD) *n*=
NP *n* = 159	4.21 (0.83)	4.25 (0.90)	3.87 (0.89)	4.28 (0.84)	4.15 (0.49)	4.10 (0.79)	4.13 (0.81)
RN *n* = 1 965	4.00 (0.79)	4.08 (0.58)	4.01 (0.84)	3.99 (0.71)	3.88 (0.96)	4.04 (0.75)	4.00 (0.79)
LPN *n* = 1 286	3.83 (0.87)	3.81 (0.82)	3.83 (0.93)	3.78 (0.83)	3.76 (0.92)	3.75 (0.78)	3.80 (0.87)
RPN *n* = 200	–	–	–	3.81 (0.95)	3.90 (0.68)	4.00 (1.10)	3.84 (0.89)
Total sample *N* = 3 610	3.93 (0.84)	4.00 (0.71)	3.92 (0.88)	3.89 (0.81)	3.84 (0.91)	4.01 (0.76)	3.92 (0.83)

Likert scale had a range of 1–5, with a higher number representing greater satisfaction

### Why nurses work in rural communities

When examining recruitment, community- and practice-related reasons (e.g., location) were most likely to be cited as reasons for coming to one’s primary work community (Table 8). The top three recruitment factors among all nurses were location of community (55.7%), interest in the practice setting (53.3%), and income (45.1%).

**Table 8:** Reasons for coming to work in primary work community by region of primary employment

Came to work in primary work community for the following reasons	Atlantic *n* (%)	QC *n* (%)	ON *n* (%)	MB/SK *n* (%)	AB/BC *n* (%)	Territories *n* (%)	Total *N* (%)
Location of community	530 (56.6)	122 (41.1)	240 (59.0)	512 (62.1)	376 (59.2)	278 (46.6)	2 058 (55.7)
Interest in practice setting	490 (52.3)	105 (35.4)	215 (52.8)	438 (53.2)	332 (52.3)	391 (65.6)	1 971 (53.3)
Income	463 (49.4)	81 (27.3)	161 (39.6)	364 (44.2)	231 (36.4)	368 (61.7)	1 668 (45.1)
Family or friends	409 (43.6)	184 (62.0)	178 (43.7)	397 (48.2)	241 (38.0)	144 (24.2)	1 553 (42.0)
Lifestyle	342 (36.5)	146 (49.2)	147 (36.1)	273 (33.1)	255 (40.2)	280 (47.0)	1 443 (39.0)
Flexibility of work	267 (28.5)	61 (20.5)	119 (29.2)	249 (30.2)	175 (27.6)	196 (32.9)	1 067 (28.9)
Benefits	324 (34.6)	107 (36.0)	95 (23.3)	196 (23.8)	135 (21.3)	196 (32.9)	1 053 (28.5)
Advanced practice opportunities	184 (19.6)	36 (12.1)	67 (15.5)	151 (18.3)	110 (17.3)	293 (49.2)	841 (22.8)
Spouse employment/transfer	148 (15.8)	37 (12.5)	79 (19.4)	209 (25.4)	156 (24.6)	115 (19.3)	744 (20.1)
Career advancement	186 (19.9)	43 (14.5)	62 (15.2)	131 (15.9)	100 (15.7)	188 (31.5)	710 (19.2)
Others	35 (3.7)	10 (3.4)	19 (4.7)	31 (3.8)	34 (5.4)	17 (2.9)	146 (4.0)
Total sample	937	297	407	824	635	596	3 696

This survey question was “mark all that apply,” so percentages will not add to 100

When analyzing by nurse type (Additional file 4: Table S3a–d), the top recruitment factors among NPs were advanced practice opportunities (67.9%) and interest in the practice setting (66.7%). Among RNs and RPNs, the top recruitment factor was interest in the practice setting (54.0% and 64.5%, respectively), and among LPNs, location of the community (60.0%).

When examining retention, the most frequently cited reasons for continuing to work in one’s primary work community (Table 9) were similar to the reasons cited for coming to such communities in the first place. Among all nurses, the top retention factors were income (56.3%), interest in the practice setting (55.5%), and location of the community (54.4%).

**Table 9:** Reasons for continuing to work in primary work community by region of primary employment

Continue to work in primary work community for the following reasons	Atlantic *n* (%)	QC *n* (%)	ON *n* (%)	MB/SK *n* (%)	AB/BC *n* (%)	Territories *n* (%)	Total *N* (%)
Income	543 (58.3)	108 (36.4)	203 (50.1)	468 (56.7)	332 (52.6)	418 (70.8)	2 072 (56.3)
Interest in practice setting	484 (51.9)	135 (45.5)	215 (53.1)	456 (55.3)	357 (56.6)	394 (66.8)	2 041 (55.5)
Location of community	518 (55.6)	119 (40.1)	224 (55.3)	499 (60.5)	376 (59.6)	265 (44.9)	2 001 (54.4)
Family or friends	492 (52.8)	185 (62.3)	213 (52.6)	449 (54.4)	315 (49.9)	231 (39.2)	1 885 (51.2)
Lifestyle	365 (39.2)	153 (51.5)	164 (40.5)	322 (39.0)	291 (46.1)	306 (51.9)	1 601 (43.5)
Flexibility of work	312 (33.5)	79 (26.6)	150 (37.0)	318 (38.5)	229 (36.3)	274 (46.4)	1 362 (37.0)
Benefits	368 (39.5)	107 (36.0)	127 (31.4)	281 (34.1)	209 (33.1)	234 (39.7)	1 326 (36.0)
Advanced practice opportunities	151 (16.2)	32 (10.8)	56 (13.8)	133 (16.1)	85 (13.5)	258 (43.7)	715 (19.4)
Spouse employment/transfer	126 (13.5)	28 (9.4)	64 (15.8)	166 (20.1)	130 (20.6)	101 (17.1)	615 (16.7)
Career advancement	112 (12.0)	48 (16.2)	38 (9.4)	93 (11.3)	70 (11.1)	161 (27.3)	522 (14.2)
Others	34 (3.6)	7 (2.4)	14 (3.5)	27 (3.3)	32 (5.1)	18 (3.1)	132 (3.6)
Total sample	932	297	405	825	631	590	3 680

This survey question was “mark all that apply,” so percentages will not add to 100

A greater percentage of LPNs and RPNs identified income as a retention factor than as a recruitment factor (Additional file 5: Table S4a–d). In addition, the top retention factor among NPs and RNs was an interest in the practice setting (69% and 59.1%, respectively); among LPNs, location of the community (58.7%) and income (58.2%); and among RPNs, income (59.5%).

## Discussion

In Canada, the proportion of regulated nurses (RNs, NPs, LPNs, RPNs) in rural and remote areas continues to fall short of the regional share of the population [1, 43]. At the same time, our study shows that a considerable proportion of the nursing workforce in rural and remote Canada (32.1%) is 55 years or older compared to the nursing workforce overall (23.2%) [1]. Canada’s experience of an aging rural nursing population is consistent with that of other countries [2, 44]. The lower proportion of rural nurses in younger age groups, particularly among women, is of concern with regard to renewal of the rural nursing workforce [45, 46].

Since 2002, the entry to practice requirement for RNs in all provinces, except Quebec, and territories has been a baccalaureate degree. Consequently, there has been a substantial increase in the percentage of rural RNs with baccalaureate education, from 27% in the RRNI study [47] to 50% in this RRNII study. The percentage of RNs with baccalaureate education varies among the regions. A certificate in rural and remote nursing was held by more nurses in BC and the territories, where these programs are offered.

Casualization of the nursing workforce has been a continuing concern in Canada that has particular ramifications in rural areas [32]. This study found that of all nurse types, NPs were the most likely to hold full-time permanent nursing positions, LPNs to hold part-time permanent positions, and RNs and LPNs to hold casual nursing positions. The percentage of rural RNs in full-time positions has not changed substantially over the last decade; it remains at about 52% overall, although it has increased in some provinces (e.g., Ontario) due to provincial policy [32].

It has long been known that for physicians, growing up in a rural community is associated with working in a rural community [5]; however, there has been little evidence specifically about nurses. This RRNII study shows that over two thirds of nurses who worked in communities of under 10 000 also grew up in communities of that size, similar to rural RNs in the RRNI study [29].

All types of nurses were satisfied with current nursing practice regardless of size of community or region of employment, but satisfaction was higher among NPs and RNs than among LPNs and RPNs. As satisfaction with practice has been found to be an important predictor of rural nurses’ intention to leave their position [19], future analyses of nurse satisfaction would need to include scope of practice, type of workplace, and engagement with interprofessional teams along with practice demands and resources in rural settings.

Nurses in this study reported that they continued to work in their primary work communities for mainly the same reasons they came to the communities in the first place: location, interest in the practice setting, and income. The ranking of these factors varied across regions and nurse type, with income, for example, a more important retention factor for LPNs and RPNs. The more prescribed roles of LPNs may influence the importance of income for retention as opposed to factors such as career advancement that are more important for RN retention. Further examination of recruitment and retention factors will be undertaken in a future analysis focused on predictors of RNs’ intent to leave their current position. Future, more nuanced analyses of various workplace and practice factors in this large country with its varied geography and populations will be done. The analyses will be informed by discussions with the RRNII study Advisory Team about workplace demands, legislation, organizational, and community challenges across the provinces and territories. As a result of this dialogue, it may be possible to identify potentially workable approaches to enhance recruitment and retention of nurses in rural and remote communities.

### Strengths and limitations

This study is the first survey of all types of nurses in the regulated nursing workforce in rural and remote Canada. It achieved an overall response rate of 40%, but the response rate varied across nurse types and provinces/territories. It is unknown whether the mixed options for survey response enhanced or harmed the response rate. The questionnaire was iteratively designed with nursing policy-makers and planners, who ensured the inclusion of important issues for Canadian rural and remote nurses. Although the questionnaire was lengthy, the use of newly developed and standardized scales and the opportunity to compare findings with the RRNI study were positive.

## Conclusions

A comprehensive survey of NPs, RNs, LPNs, and RPNs working in rural and remote areas of all provinces and territories in Canada was undertaken in 2014 and 2015 (RRNII). This survey expanded upon the survey carried out in 2001–2002 with RNs across Canada as part of the RRNI study. The current survey has identified community, workplace, and personal factors that are important to understand about the rural and remote nursing workforce. For example, we have identified that the majority of nurses working in rural and remote communities also grew up in rural communities.

As Malatzky and Bourke [48] note, new discourses are needed to communicate the strengths and benefits of rural communities, to maximize the possibilities of increasing and stabilizing the health workforce in those communities. Greater knowledge about each type of nurse within the regulated nursing workforce in rural and remote communities as generated through this study, coupled with knowledge about their perceptions of their practice and the context of their work, will inform such discourses. With greater knowledge, there can be renewed, more positive discourses about the strengths and possibilities within rural and remote nursing practice. As well, more focused attention can be given to those factors that support practice for all types of nurses in various regions.

## Additional files

Additional file 1: Figure S1. Conceptual framework.

Additional file 2: Table S1. Priority areas and research objectives.

Additional file 3: Table S2. Employment status by nurse type and region of primary employment.

Additional file 4: Table S3a. NP, reasons for coming to work in primary work community by region of primary employment. **Table S3b.** RN, reasons for coming to work in primary work community by region of primary employment. **Table S3c.** LPN, reasons for coming to work in primary work community by region of primary employment. **Table S3d.** RPN, reasons for coming to work in primary work community by region of primary employment.

Additional file 5: Table S4a. NP, reasons for continuing to work in primary work community by region of primary employment. **Table S4b.** RN, reasons for continuing to work in primary work community by region of primary employment. **Table S4c.** LPN, reasons for continuing to work in primary work community by region of primary employment. **Table S4d.** RPN, reasons for continuing to work in primary work community by region of primary employment.

## Abbreviations

AB: Alberta
ANOVA: Analysis of variance
BC: British Columbia
FT: Full time
HSD: Tukey’s honestly significant difference
LPN: Licensed practical nurse (registered practical nurse in Ontario)
MB: Manitoba
NB: New Brunswick
NL: Newfoundland and Labrador
NP: Nurse practitioner
NS: Nova Scotia
NT: Northwest Territories
NU: Nunavut
ON: Ontario
PE: Prince Edward Island
PT: Part time
QC: Quebec
RN: Registered nurse
RPN: Registered psychiatric nurse
RRNI: The Nature of Nursing Practice in Rural and Remote Canada study
RRNII: Nursing Practice in Rural and Remote Canada study
SK: Saskatchewan
YT: Yukon

## Footnotes

1 The questionnaire is available from the authors;

2 To maintain confidentiality of the participants, data is reported in tables when there is a minimum cell size of five. When this condition is not met, cells are suppressed;

3 Nurse types were grouped—NPs and RNs; LPNs and RPNs—in order to achieve substantial enough sample sizes to conduct significance tests and post hoc comparisons.
