# Supplementary figures and images for: Nurses who work in rural and remote communities in Canada: a national survey
Source: Hum Resour Health. 2017 May 23;15:34. doi: 10.1186/s12960-017-0209-0 (PMC5442670; doi:10.1186/s12960-017-0209-0)

**Figure S1. Conceptual Framework**


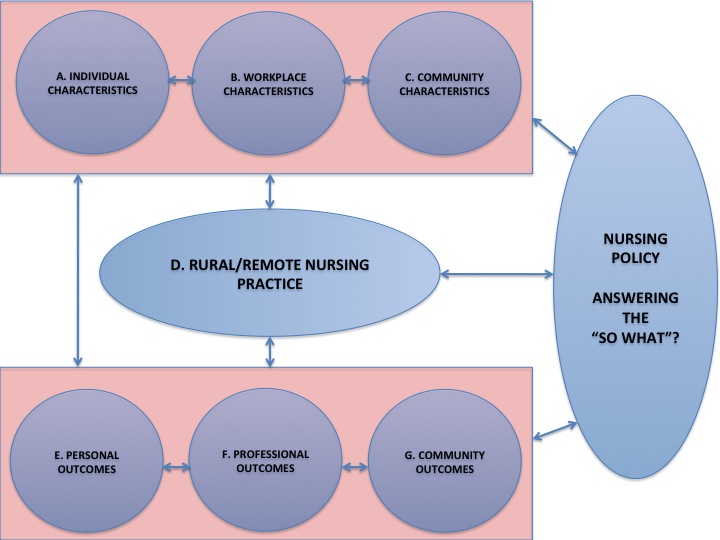

Supplement: Supplementary file 1 — Conceptual framework. [file 12960_2017_209_MOESM1_ESM.docx]
