# Supplementary material for: Nurses who work in rural and remote communities in Canada: a national survey
Source: Hum Resour Health. 2017 May 23;15:34. doi: 10.1186/s12960-017-0209-0 (PMC5442670; doi:10.1186/s12960-017-0209-0)
Supplement: Supplementary file 2 — Priority areas and research objectives. [file 12960_2017_209_MOESM2_ESM.docx]

**Nursing Practice in Rural and Remote Canada II**

**Research Objectives**

There are two central research questions:

- What is the nature of nursing practice in rural and remote Canada?
- How can the access to nursing care in rural and remote Canada be enhanced?

Table S1. Priority Areas and Research Objectives

| Priority Areas | Research Objectives |
| --- | --- |
| 1. Nursing roles and functions, including engagement of nurses with PHC | 1. Describe and compare the roles and functions of nurses practicing in various work environments and with differing scopes of practice to determine how roles are enacted in rural/remote communities |
|  | 2. Describe the engagement of nurses with PHC in rural/remote communities |
| 2. Recruitment | 3. Examine what contributes to the recruitment of nurses to rural/remote practice |
| 3. Retention | 4. Develop a predictive model for retention of nurses in rural/remote practice |
| 4. Preparedness for practice | 5. Develop a predictive model for preparedness for nursing practice in rural/ remote communities |
| 5. Implications for knowledge translation (KT) and policy | 6. Determine changes in the nature of rural/remote nursing practice over the last decade |
|  | 7. Determine how nurses in rural/remote Canada can best be prepared, recruited, and supported in their work |
|  | 8. Identify priorities for organizational support, policy support and basic and ongoing education of nurses in rural/remote Canada |
|  | 9. Contribute to policy discussions on nurses' practice, recruitment, retention, and education in rural/remote Canada |
