# Supplementary material for: Nurses who work in rural and remote communities in Canada: a national survey
Source: Hum Resour Health. 2017 May 23;15:34. doi: 10.1186/s12960-017-0209-0 (PMC5442670; doi:10.1186/s12960-017-0209-0)
Supplement: Supplementary file 3 — Employment status by nurse type and region of primary employment. [file 12960_2017_209_MOESM3_ESM.docx]

| Table S2. ***Employment Status by Nurse Type and Region of Primary Employment*** | | | | | | | | |
| --- | --- | --- | --- | --- | --- | --- | --- | --- |
| **Nursing**  **Employment Status** | **Atlantic**  **n** (%) | **QC**  **n** (%) | **ON**  **n** (%) | **MB/SK**  **n** (%) | **AB/BC**  **n** (%) | **Territories**  **n** (%) | **Total**  **N** (%) |  |
| FT Permanent | 23 (76.7) | 20 (83.3) | 26 (83.9) | 19 (76.0) | 14 (70.0) | 20 (64.5) | 122 (75.8) |  |
| PT Permanent | - | 5 (20.8) | - | 5 (20.0) | - | - | 24 (14.9) |  |
| Job Share | - | - | - | - | - | - | - |  |
| Casual | - | - | - | - | - | - | 10 (6.2) |  |
| Contract/Term | - | - | - | - | - | 5 (16.1) | 10 (6.2) |  |
| **Total NP Sample** | **30** | **24** | **31** | **25** | **20** | **31** | **161** |  |
| FT Permanent | 312 (63.3) | 103 (59.2) | 118 (57.6) | 164 (44.4) | 114 (35.2) | 261 (52.4) | 1072 (52.0) |  |
| PT Permanent | 114 (23.1) | 59 (33.9) | 63 (30.7) | 140 (37.9) | 155 (47.8) | 76 (15.3) | 607 (29.4) |  |
| Job Share | - | - | - | 9 (2.4) | - | 15 (3.0) | 31 (1.5) |  |
| Casual | 64 (13.0) | 8 (4.6) | 29 (14.1) | 69 (18.7) | 61 (18.8) | 109 (21.9) | 340 (16.5) |  |
| Contract/Term | 13 (2.6) | 5 (2.9) | 7 (3.4) | 15 (4.1) | 15 (4.6) | 79 (15.9) | 134 (6.5) |  |
| **Total RN Sample** | **493** | **174** | **205** | **369** | **324** | **498** | **2063** |  |
| FT Permanent | 290 (67.4) | 39 (34.5) | 113 (62.1) | 115 (38.7) | 94 (37.2) | 54 (68.4) | 705 (52.1) |  |
| PT Permanent | 85 (19.8) | 71 (62.8) | 63 (34.6) | 133 (44.8) | 105 (41.5) | 13 (16.5) | 470 (34.7) |  |
| Job Share | - | - | - | - | - | - | 9 (0.7) |  |
| Casual | 63 (14.7) | - | 13 (7.1) | 66 (22.2) | 68 (26.9) | 11 (13.9) | 223 (16.5) |  |
| Contract/Term | 9 (2.1) | - | - | 5 (1.7) | 5 (2.0) | - | 24 (1.8) |  |
| **Total LPN Sample** | **430** | **113** | **182** | **297** | **253** | **79** | **1354** |  |
| FT Permanent | - | - | - | 93 (64.6) | 33 (61.1) | - | 128 (62.7) |  |
| PT Permanent | - | - | - | 39 (27.1) | 19 (35.2) | - | 58 (28.4) |  |
| Job Share | - | - | - | - | - | - | - |  |
| Casual | - | - | - | 18 (12.5) | - | - | 25 (12.3) |  |
| Contract/Term | - | - | - | - | - | - | - |  |
| **Total RPN Sample** | **-** | **-** | **-** | **144** | **54** | **6** | **204** |  |
