# Supplementary material for: Nurses who work in rural and remote communities in Canada: a national survey
Source: Hum Resour Health. 2017 May 23;15:34. doi: 10.1186/s12960-017-0209-0 (PMC5442670; doi:10.1186/s12960-017-0209-0)
Supplement: Supplementary file 4 — NP, reasons for coming to work in primary work community by region of primary employment. Table S3b. RN, reasons for coming to work in primary work community by region of primary employment. Table S3c. LPN, reasons for coming to work in primary work community by region of primary employment. Table S3d. RPN, reasons for coming to work in primary work community by region of primary employment. [file 12960_2017_209_MOESM4_ESM.zip › Supplemental Table 3dR1.docx]

| Table S3d. ***RPN, Reasons for Coming to Work in Primary Work Community by Region of Primary Employment*** | | | | | | | |
| --- | --- | --- | --- | --- | --- | --- | --- |
| **Came to work in primary work community for the following reasons** | **Atlantic**  **n** (%) | **QC**  **n** (%) | **ON**  **n** (%) | **MB/SK**  **n** (%) | **AB/BC**  **n** (%) | **Territories**  **n** (%) | **Total**  **N** (%) |
| Advanced practice opportunities | - | - | - | 25 (17.4) | 8 (15.1) | - | 37 (18.2) |
| Benefits | - | - | - | 50 (34.7) | 17 (32.1) | - | 70 (34.5) |
| Career advancement | - | - | - | 27 (18.8) | 8 (15.1) | - | 36 (17.7) |
| Family or friends | - | - | - | 62 (43.1) | 20 (37.7) | - | 83 (40.9) |
| Flexibility of work | - | - | - | 45 (31.3) | 16 (30.2) | - | 65 (32.0) |
| Interest in practice setting | - | - | - | 97 (67.4) | 29 (54.7) | 5 (83.3) | 131 (64.5) |
| Lifestyle | - | - | - | 50 (34.7) | 11 (20.8) | 5 (83.3) | 66 (32.5) |
| Location of community | - | - | - | 92 (63.9) | 24 (45.3) | - | 119 (58.6) |
| Income | - | - | - | 67 (46.5) | 24 (45.3) | - | 95 (46.8) |
| Spouse employment/transfer | - | - | - | 32 (22.2) | 8 (15.1) | - | 40 (19.7) |
| Other | - | - | - | 8 (5.6) | - | - | 12 (5.9) |
| **Total RPN Sample** | **-** | **-** | **-** | **144** | **53** | **6** | **203** |
| Note. *This survey question was ‘mark all that apply’, so percentages will not add to 100.* | | | | | | | |
