# Supplementary material for: Nurses who work in rural and remote communities in Canada: a national survey
Source: Hum Resour Health. 2017 May 23;15:34. doi: 10.1186/s12960-017-0209-0 (PMC5442670; doi:10.1186/s12960-017-0209-0)
Supplement: Supplementary file 5 — NP, reasons for continuing to work in primary work community by region of primary employment. Table S4b. RN, reasons for continuing to work in primary work community by region of primary employment. Table S4c. LPN, reasons for continuing to work in primary work community by region of primary employment. Table S4d. RPN, reasons for continuing to work in primary work community by region of primary employment. [file 12960_2017_209_MOESM5_ESM.zip › Supplemental Table 4dR1.docx]

| Table S4d. ***RPN, Reasons for Continuing to Work in Primary Work Community by Region of Primary Employment*** | | | | | | | |
| --- | --- | --- | --- | --- | --- | --- | --- |
| **Continue to work in primary work community for the following reasons** | **Atlantic**  **n** (%) | **QC**  **n** (%) | **ON**  **n** (%) | **MB/SK**  **n** (%) | **AB/BC**  **n** (%) | **Territories**  **n** (%) | **Total**  **N** (%) |
| Advanced practice opportunities | - | - | - | 18 (12.4) | 5 (9.3) | - | 25 (12.2) |
| Benefits | - | - | - | 62 (42.8) | 18 (33.3) | - | 82 (40.0) |
| Career advancement | - | - | - | 20 (13.8) | - | - | 24 (11.7) |
| Family or friends | - | - | - | 75 (51.7) | 25 (46.3) | - | 102 (49.8) |
| Flexibility of work | - | - | - | 59 (40.7) | 14 (45.9) | - | 77 (37.6) |
| Interest in practice setting | - | - | - | 84 (57.9) | 26 (48.1) | 6 (100.0) | 116 (56.6) |
| Lifestyle | - | - | - | 59 (40.7) | 17 (31.5) | - | 80 (39.0) |
| Location of community | - | - | - | 80 (55.2) | 28 (51.9) | - | 109 (53.2) |
| Income | - | - | - | 87 (60.0) | 29 (53.7) | 6 (100.0) | 122 (59.5) |
| Spouse employment/transfer | - | - | - | 29 (20.0) | 9 (16.7) | - | 38 (18.5) |
| Other | - | - | - | 8 (5.5) | - | - | 12 (5.9) |
| **Total RPN Sample** | **-** | **-** | **-** | **145** | **54** | **6** | **205** |
| Note. *This survey question was ‘mark all that apply’, so percentages will not add to 100.* | | | | | | | |
